# The Maze of APP Processing in Alzheimer’s Disease: Where Did We Go Wrong in Reasoning?

**DOI:** 10.3389/fncel.2015.00186

**Published:** 2015-05-28

**Authors:** Ming Chen

**Affiliations:** ^1^Aging Research Laboratory, Research and Development Service, Bay Pines VA Healthcare System, Bay Pines, FL, USA; ^2^Department of Molecular Pharmacology and Physiology, University of South Florida College of Medicine, Tampa, FL, USA

**Keywords:** Alzheimer’s, amyloid, tau, calcium, presenilin

## Abstract

Why has Alzheimer’s disease (AD) remained a conundrum today? The main reason is the stagnation in understanding the origins of plaques and tangles. While they are widely thought to be the products of the “aberrant” pathways, we believe that plaques and tangles result from natural aging. From this new perspective, we have proposed that age-related inefficiency of α-secretase is the underpinning for Aβ overproduction. This view contrasts sharply with the current doctrine that Aβ overproduction is the product of the “overactivated” β- and γ-secretases. Following this doctrine, it has been claimed that the two secretases are “positively identified” and that their inhibitors have “successfully reduced Aβ levels.” But, why have these studies not led to the understanding of AD or successful clinical trials? And if so, where did they go off course in reasoning? These questions may touch the basics of biological science and must be answered. In this paper, I dissected several prevailing assumptions and some influential reports with an attempt to trace the origins of the conundrum. This work led me to an original model for Aβ overproduction and also to a serious question: given the universal knowledge that boosting α-secretase reduces Aβ, a straightforward highway for intervention, then why is there such an obsession on “inhibiting β- and γ-secretases,” a much more costly and twisting road even if possible? This issue requires the attention of policymakers and all researchers. I therefore call for a game change in AD study.

## Introduction

Amyloid plaques and neurofibrillary tangles characterize aging human brains, and they are also accompanying or correlating with the progression of late-onset sporadic Alzheimer’s disease (sAD; also known as senile dementia or LOAD) in some elderly. As such, understanding their mechanism of formation is essential for understanding sAD, but this mechanism has remained elusive after intensive studies for decades (Masters and Beyreuther, 2006; O’Brien and Wong, 2011; Nelson et al., 2012).

Because plaques and tangles exist in essentially all elderly, we believe that they are the products of aging or age-related insufficient proteolysis. From this new perspective, we have proposed that an inefficient α-secretase in the normal processing of amyloid-β precursor protein (APP) is primarily responsible for Aβ overproduction, and is also the rational drug target for intervention. This view is in line with the knowledge that boosting APP α-processing reduces Aβ both *in vitro* and *in vivo* (Chen, 1997; Chen and Fernandez, 2001).

This knowledge, however, has been largely ignored by the field where a dominant theory today is that Aβ overproduction is the result of “overactivation” of “rate-limiting” β- and γ-secretases. Following this doctrine, it has been reported that the two secretases are “positively identified” and their inhibitors have “successfully reduced Aβ” (De Strooper et al., 2010; Selkoe, 2011). These studies, however, have never convinced the medical community as a whole, nor have been corroborated by the clinical trial results (Chen and Nguyen, 2014). Thus, sAD has remained a major scientific enigma of this century.

How has this happened? We have pointed out that redefining *senile dementia* (sAD) as a discrete/curable “disease” by the National Institute on Aging (NIA) is the initial problem, which mandates a “pathogenic” pathway, “independent of aging,” to be found in a *senile disorder* that apparently results from population aging (Chen et al., 2011a).

But, the current “β- and γ-secretases” doctrine has been promoted by leading researchers, supported by mountain loads of experimental results and published in top-notch journals. How, then, can it be unconvincing to the general medical community and, if so, where did it go off track in reasoning? In this paper, I dissected several prevailing assumptions and some influential reports with an attempt to trace the origins of the conundrum.

## An Overly Stretched Picture of APP Processing

**Figure 1:**
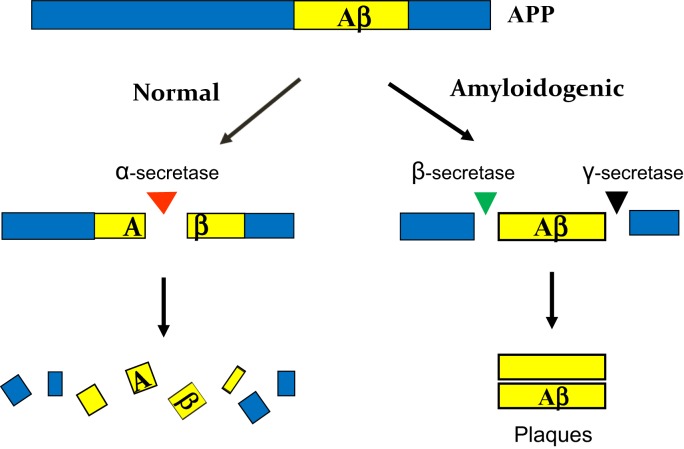
**A commonly used picture for APP processing**. It conveys a general idea for two pathways and their end results. But the picture has been overstretched in three key points: (i) the two pathways are thought to occur at the same time, so they would be “independent” of each other and “compete” for APP; (ii) as such, Aβ overproduction may be explained by an “overactivated” amyloidogenic pathway; and (iii) the three secretases in question are each depicted in a singular form, so they all seem to be identifiable. These overstretches have led to many far-reaching consequences in sAD study.

It appears that many current problems are rooted in a commonly used picture, which sketches the two pathways in APP processing (Figure 1; among many similar ones). This picture, in concept, rightly brings us to the core issues: the source of Aβ and the process that produces it. It also vividly names the three proteases involved as “secretase,” pointing to their unique roles in protein secretion rather than degradation as most proteases do. Perhaps owing to these merits, the picture has been used by almost all investigators as a roadmap (ourselves included).

But, a truth is true only within its defined boundaries, and any overstretching or over interpreting, even by a razor-thin deviation off the boundaries, can sometimes make it a fallacy with profound consequences. Indeed, upon a re-examination with caution, it came to my attention that the picture has been overstretched in at least three key aspects.

(1) The two pathways, as the way they are drawn, can be taken to imply that they occur *at the same time*, as such are *independent* of each other. These implications, under the mandate of NIA for a “pathogenic” pathway, have grown over the years from trickle to flow and eventually to a full-blown doctrine. Which posits that the amyloidogenic pathway is exactly the one that NIA is looking for and thus only it, not α-pathway, is worth to study (Selkoe, 2005; and many studies on this pathway only) – akin to a pathogen-induced pathway in patients only, not in healthy individuals.
(2) If the two pathways occur at the same time, then they would be expected to *compete* for APP. It thus seems plausible to assume that the best way to explain Aβ overproduction is by an “overactivation” or “overexpression” of β- and γ-secretases, which liberate Aβ out of APP before α-secretase can act. This reasoning has cultivated the notion that β- and γ-secretases are “rate-limiting” in the process, thus inhibiting them would reduce Aβ “at its root” – age-related changes in APP normal catabolism can be neglected.
(3) The three proteases in question are commonly depicted in *singular form*, α-, β-, and γ-“secretase,” respectively. This was miniscule, but today it is the cornerstone for enormous efforts and resources devoted to the identification of β- and γ-secretases and many clinical trials on their inhibitors. All it is because the singular term carries with it a connotation that there is *only one* protease releasing Aβ at its either end – its *singularity*, a formidable premise.

Such overstretches of the picture, intentional or unintentional, have pushed its boundaries to extreme and constituted the default points for further ramifications in numerous studies. These studies reinforce, rationalize, and defend one other and together they have generated numerous papers, patents and excitements. But why has sAD remained an enigma? It may be because, at least in part, they have not explained several hard questions.

(a) *By what molecular mechanism* can proteases be overactivated or overexpressed during normal and early aging?
(b) Even if they are somehow overactivated or overexpressed by an as-yet-unknown mechanism, but where do the additional full-length APPs, the necessary precursors for overproducing Aβs, come from?
(c) Even if additional APPs are somehow available and Aβ is generated somehow by overactivated β-/γ-secretases, but how can it stay and resist the indiscriminate attacks by many non-specific proteases in the brain? (This is the most bizarre puzzle in sAD that must be explained by any models).
(d) Why does this “protease-resistance” not happen to most other proteins? This also questions the “protein misfolding” model, since no aberrant structures have ever been found in Aβ, tau or other co-deposited proteins to make them selectively prone to misfolding (Chen and Nguyen, 2014). Such structures may not be found in the future, since they imply that plaques and tangles would be inherited or unmodifiable – in fact, they can be easily modified by various common approaches such as physical exercises (García-Mesa et al., 2011).

## A New Picture for APP Processing

**Figure 2:**
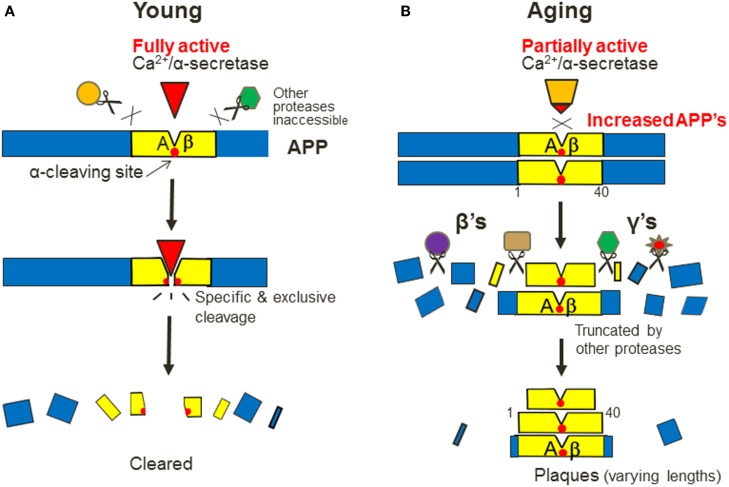
**A new model for the mechanism of A*β* overproduction in sporadic AD**. It emphasizes three key points: (1) the “two pathways” do not occur at the same time, but years apart, in young and aging **(A,B)**. By this view, there is only one, α-processing pathway that exists in two functional states; and only after it becomes inefficient during aging, can the “amyloidogenic” pathway be “overactivated”; (2) α-secretase is a Ca^2+^-dependent protease that recognizes APP in a specific and exclusive manner, like a “ligand–receptor” pair **(A)**. So after it becomes inefficient, full-length APPs would not be attacked by any other proteases at the same site, so they will accumulate transiently as the additional precursors to overproduce Aβs; and (3) the intact APPs are then truncated by many non-specific proteases, which are depicted as β’s and γ’s in plural forms, thereby producing Aβs in varying lengths **(B)**.

These questions may have touched the basics of biological principles and casted doubts on the scientific bases of the “β-/γ-secretases” doctrine, thereby calling for fundamentally different and innovative ideas to explain Aβ overproduction. To this end, we have undertaken a bottom-up analysis of the basic issues in sAD and, as a result, proposed an original model from a new perspective of “aging and energy-Ca^2+^ deficits” (Chen and Fernandez, 1999; Chen and Nguyen, 2014). Now, I elaborate the model by emphasizing three key points (Figure 2).

First and foremost, the “two pathways” in sAD do not occur at the same time, but years apart in two distinctive life stages: *young* and *aging* (Figures 2A,B). By this view, there would be no “two pathways” anymore, rather there is only one normal pathway that exists in two functional states: only after it is inefficient during aging, can the “amyloidogenic” pathway become prominent – or seemingly “overactivated” – as a result. Hence, the only reasonable way to reduce Aβ plaques is by activating α-processing, just like activating lipid degradation reduces cholesterol plaques.

Second, α-secretase is a Ca^2+^-dependent protease that recognizes APP in a specific and *exclusive* manner, like a ligand–receptor duo (Figure 2A; the reasons for this unusual relationship were discussed previously; Chen and Nguyen, 2014). During aging, α-secretase is inefficient as a result of energy and Ca^2+^ signaling deficits. This would lead to a transient increase of full-length APPs, which are not attacked by any other proteases at the α-site but are alternatively truncated by many non-specific proteases at other sites. So their fragments surrounding the α-site will be spared and deposited as Aβs with varying lengths (Figure 2B). Thus, Aβ is protease-resistant but most other proteins are not (i.e., only the substrates of Ca^2+^-dependent proteases will deposit during aging).

Third, as many proteases can be involved in the later stage of Aβ genesis, I depict them in *plural forms*, i.e., “β’s” and “γ’s,” which stand for “β-site APP cleaving enzymes” (BACEs, as commonly used) and “γ-site APP cleaving enzymes,” respectively. As unregulated proteases, their products can increase only after the substrates have increased (Figure 2B).

## Has “β-Secretase” been “Positively Identified”?

The new model thus suggests that “identification of β- or γ-secretase” as a single enzyme entity is a problematic concept in inception, since Aβs appear to be similar to many other protein fragments or peptides in our body that are randomly produced by various proteases.

Nevertheless, three prominent studies have claimed that they identified a protease that is responsible for the cleavage of APP at the Aβ1 position (“β-site”) and it also displays other presumed features of the putative enzyme, thus called it “β-secretase” (Hussain et al., 1999;Vassar et al., 1999;Yan et al., 1999). But caveats remain.

(a) The claim apparently rests on the assumptions that there is only one protease clipping the Aβ1-site and that all Aβs have uniform N-termini at that site. In reality, the observed N-termini of Aβs start from a range of at least 13 amino acids, which may involve the actions of other proteases (Chen and Nguyen, 2014; and references therein).
(b) Even if all Aβs start from the same site, there is still a hard task ahead: to prove that *no other proteases* can also act at that site. This task is especially necessary as the main goal of the studies is to develop inhibitors of the enzyme to reduce Aβ *in vivo*. Evidently, this goal would not be feasible if other proteases can also act at that site.
  Can these other proteases be excluded from Aβ1-site clipping? This would require testing *all proteases* in the body and excluding them one by one – a mission formidable or impossible. As a matter of fact, other β-site-cleaving proteases have been found (Evin et al., 1994; Sinha et al., 1999), disproving the “singularity” of β-secretase in concept.
  Thus, it is more correct to say that the studies have identified *one of the proteases* that together produce *one type* of Aβs (starting at Aβ1 position). This would question the use of its inhibitors to reduce the overall levels of Aβs (see below).
(c) The three studies also claim that the identified β-secretase is “membrane-bound.” Notably, such a protease would have an out-reaching limit and that limit is 12/13 amino acids from the cell surface, as two elegant studies have shown (Maruyama et al., 1991; Sisodia, 1992). The Aβ1-site is 28 amino acids from the cell surface. And also, how can a single membrane-bound protease, with its mobility hindered, generate heterogeneous N-termini of Aβs? (Compare with membrane-bound α-secretase; see below).

## Why Presenilins are Unlikely to be “γ-Secretase”

At the same time, it has also been claimed that “γ-secretase” is identified as presenilins (PS1 and PS2) with the active center at D257 or D385 in PS1 (Citron et al., 1997; Wolfe et al., 1999). Besides the required justifications for its “singularity”, “overactivation” by aging, and the heterogeneous C-termini of Aβs (ending at a range of at least 12 amino acids), this claim also faces additional questions.

**Figure 3:**
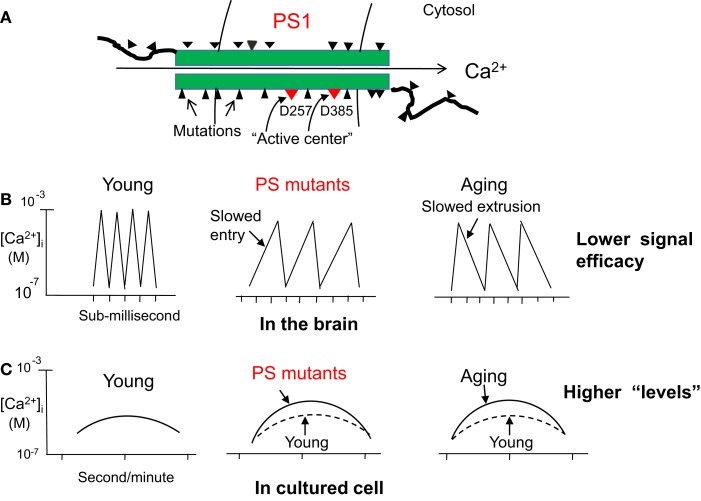
**Why presenilin 1 (PS1) is unlikely to be γ-secretase, but likely a Ca^2+^ channel**. **(A)** A simplified drawing that highlights many mutations on PS1. If it is a protease, then some mutations that are hundreds of amino acids away from the “activity center” (D257/D385) would be unlikely to affect the protease activity. However, if PS1 is a Ca^2+^ channel, then mutations anywhere on it would all disturb the super-rapid Ca^2+^ channeling process with similar consequences. **(B)** As Ca^2+^ channels, both PS1 and PS2 mutations will mostly reduce Ca^2+^ entry, not extrusion, since energy may not change in the young mutant hosts. By contrast, an energy crisis in elderly will mostly slow down Ca^2+^ extrusion, not entry, a spontaneous process driven by the ion gradient. Thus, both cases will end up in the same net result: reduced Ca^2+^ signaling potency in the brain (in sub-millisecond or microsecond). **(C)** However, if Ca^2+^ is measured in cultured cells (by second or longer time intervals), its changes caused by either mutations or aging will manifest as higher steady-state “levels.”

First, if PS1 is “γ-secretase,” then how can it be overactivated by each of its near-200 gene mutations known today (Figure 3A)? Presumably, most pathological mutations cause human diseases by loss-of-function (Kelley, 1992), so such a “gain-of-function” mechanism would be rare or minority cases. Thus, the probability that near-200 mutations all render the protease more active would be remote.

Second, more importantly, the mutant amino acids are spreading everywhere on PS1, and many of them are as far as *hundreds* of residues away from the D257 or D385 site and others are in the outside of the cell (Figure 3A). By what mechanism can they increase the protease cleavage?

Now, why do I believe that most mutations cause diseases by loss-of-function? Because (i) most mutation-caused human diseases are loss-of-function in molecular nature (e.g., Sickle cell anemia, cystic fibrosis, early onset familial hearing loss or atherosclerosis; Cotran et al., 1999); and (ii) our body is essentially an *efficient* system after a long process of evolution, so mutational changes in it are more likely to decrease, rather than increase, its functionality.

These points are especially evident in the “Ca^2+^ channels” model for PSs (Chen, 1998a). High frequency Ca^2+^ waves are by far the most sensitive and “digital” information-carrying regulator in our body (Putney, 1998). As such, Ca^2+^ channels must be highly exquisite especially for cognition, the most delicate function of humans. Thus, although the near-200 mutations spread everywhere on the PS1 channel, it is no difficult to see that they will all disturb the super-fast passage of Ca^2+^ through the channel, a process that occurs intermittently at an astonishing frequency of over a thousand times per second (sub-millisecond or microsecond) in the brain (Llinás and Moreno, 1998). Needless to say, such an extraordinarily intricate and precise process would be highly sensitive to any subtle changes in the conformation, affinity, or electrostatic charges of the channel brought by the mutant amino acids, no matter where they are located (Figure 3A). This may be why all pathogenic mutations cause similar *loss of memory function* in their hosts.

The model also suggests that PS mutations most likely slow Ca^2+^ entry, not extrusion, in each entry-extrusion cycle since the latter is energy-driven but young mutant hosts may not have energy deficiency (Figure 3B). In contrast, age-related Ca^2+^ deficit should occur mainly as slowed Ca^2+^ extrusion, thanks to the energy crisis in the elderly, but not Ca^2+^ entry, a process driven by the steep ion gradient. Thus, both cases would end up in the same net result: reduced Ca^2+^ signaling efficacy in the brain (Figure 3B), which will lead to permanent and early inactivation of α-secretase and calpain, thereby resulting in severe plaques and tangles in the young mutant hosts. However, if Ca^2+^ is measured in cultured aging cells, the reduced Ca^2+^ signaling potency would manifest as higher steady-state Ca^2+^ “levels” (Figure 3C) (Chen et al., 2011b; Nguyen et al., 2013).

There are numerous Ca^2+^ channels in the brain and they are all subject to random gene mutations, but why only mutations in PSs channels, not in any other channels, are segregated with early dementia? This is most likely because PSs channels are more important to cognition than other channels. This may allow a new look at the long-held notion that other, more abundant Ca^2+^/cation channels such as NMDA, AMPA or rynodine receptors play primary roles in cognition. So, PSs may be a more relevant tool for studying cognition.

## Have the Inhibitors of “β- or γ-Secretase” Reduced Aβ?

Despite the unanswered questions in the “β- or γ-secretase” doctrine, a considerable number of studies have nonetheless followed it and reported that their synthetic inhibitors have “successfully reduced Aβ levels,” thus spurring many high-profile clinical trials on the inhibitors. But such reports themselves have also left disturbing questions.

For example, many studies have reported that inhibiting “β-secretase”, *alone*, reduces Aβ (Evin et al., 2011). But a key implication of the studies has not been mentioned: “γ-secretase” would not be important anymore, since it is not tackled yet the Aβ level changed. On the other hand, however, many other studies have also reported that inhibiting “γ-secretase,” *alone*, reduces Aβ (Imbimbo and Giardina, 2011), thereby proving “β-secretase” being unimportant by the same token.

Apparently, such mutually conflicting studies would defy each another’s scientific values, especially since neither camp has discussed the implications of the other, an un-scientific manner. More intriguingly, none of them has thought of the changes of α-secretase, a vibrant and highly sensitive player that controls the availability of full-length APPs in any cells that produce Aβ. Can any drugs reduce cholesterol levels without affecting normal degradation of lipids?

Moreover, the release of Aβ apparently involves two sequential or simultaneous cleavages at its both ends, so how can a single inhibitor reduce it? Further, if various proteases can be involved in the release of heterogeneous Aβs, will inhibiting one of them reduce the overall levels of the peptides significantly? What if the compensatory effects of other proteases are also taken into account?

Evidently, unless these questions are answered on the basis of the established biological laws, it is perhaps premature to expect a “miracle cure” by the inhibitors.

## Why α-Secretase Can be Identified

While the singular form for “β- or γ-secretase” is problematic, I nevertheless think that it is correct for α-secretase for two reasons. First, direct sequencing of the secreted APP by α-secretase from a wide variety of cell types including insect and yeast has only found its C-terminus ending near-uniformly at Aβ15/16 (Esch et al., 1990; Anderson et al., 1991; Wang et al., 1991; Lowery et al., 1991; Ramabhadran et al., 1993; Zhang et al., 1994), despite other minor species being detected by highly sensitive methods. Second, theoretically, the “double anchorage” of both APP and α-secretase in the membrane, which explains the site-specific APP α-cleavage, may not allow additional α-secretases there, a redundant system.

Therefore, despite the proposed “two α-secretases,” constitutional and stimulated (Lammich et al., 1999), it is my opinion that there is only one α-secretase in APP processing, but exists in two functional states: basal and stimulated *in vitro*, or efficient and inefficient *in vivo* (Figure 2). Such experimental or age-related changes perhaps also occur in other lifeline enzymes.

At the same time, the Ca^2+^-dependent nature of α-secretase, if established, would preclude most other proteases in the repertoire as its reasonable candidates. I therefore believe that α-secretase would be identified in the end.

## What is Behind the Obsession for “β-/γ-Secretases”?

Besides these scientific issues, I also noticed that there is a long-existing and black-and-white question beyond scholarly debates. This question should alert all of us.

Given the universal knowledge that boosting α-secretase reduces Aβ, a rational and straightforward highway for intervention and, particularly, that a full spectrum of effective agents to do so have long been known (Chen, 1997; Mattson, 1997), why, then, is there such an obsession and fanatic commitment to “inhibiting β- and γ-secretases,” a much more costly and biologically twisting road even if possible, in the first place?

A shadowy motive may not be ruled out: only the latter road brings more patents, sales and wealth, a “dark energy” that has been driving AD research for too long and too passionately. Making wealth aside, why knowingly skew research direction at the expense of science and patients’ interests, even though it fits NIA perception? A soul-searching by policymakers and some elite researchers may be helpful.

For-profit studies have played important roles in medical history, especially in drug *development* stages. But a worrisome reality in AD study is that they have played a heavy role in the *fact-finding* stage, a subtle and curiosity-driven process that can be unnoticeably tilted off course by non-science forces at the key turning points (e.g., excessive aging or “*bona fide* disease”; boosting brain health or “inhibiting toxins”). Meanwhile, such forces may have also played a role in the making of the dogmatic status of the *unproven* “Ca^2+^ overload” hypothesis (Khachaturian, 1994) (calcium channel blocker sales).

No wonder why many commonsense issues can remain controversial after 40 years (e.g., normal or “abnormal”; targeting aging process or “cell-death”; Ca^2+^ deficiency or “excess” in aging) – in the deepest roots, they may reflect, at least in part, the conflicts between science and vested interests.

## A Critical Decision for NIA

By recalling the process in which the field has shifted the study focus from α-secretase to “β-/γ-secretases” over the years, I noticed that the transition has happened perhaps in the following footsteps.

(i) It has been established that α-secretase is sensitively regulated by many signal pathways (e.g., glutamatergic, cholinergic, ERK/MARK-, PKC-, energy related- and IP3-pathways, among others). However, intervention design has stagnated, because it is unpractical to target these many pathways at once. At the same time, no common denominator for these pathways has been validated and accepted.
(ii) This study impasse would allow the field to drift towards metalloproteases – another dead end since unregulated proteases, in the end, are unlikely to be re-activated by common approaches for intervention. And if they are α-secretase, then it may not explain why Aβ is protease-resistant (Chen and Nguyen, 2014).
(iii) These gridlocks thus would set the stage for “β-/γ-secretases” studies to boom. Under the powerful influences of vested interests and NIA perceptions, such studies have quickly overwhelmed the landscape and, meanwhile, inhibited scholarly criticisms, the last line of defense for the integrity of science.

Now, comparison of the three study directions suggests that targeting α-secretase, a *regulated* enzyme, is the only rational paradigm for intervention. However, a persistent obstacle here is the lack of a *common regulatory mechanism* for the enzyme, the bottleneck to the study progress. In this regard, I proposed the concept of “Ca^2+^-dependent α-secretase” (Chen, 1997), or “energy-Ca^2+^” as the common drug target, a proposal that is supported by a solid and growing body of experimental evidence and, especially, it has an unusual explanatory power for the sAD features (Chen and Nguyen, 2014).

However, metalloproteases and “β-/γ-secretases” have also been widely said to be “regulated” because they, too, are responsive to the signal pathways (Imbimbo and Giardina, 2011; Lichtenthaler, 2012). Since Ca^2+^ is perhaps the only true signal transduction-controlled protease regulator known today, I think that metalloproteases and other proteases are not regulated, but *affected*, by the activated signal pathways, which will trigger a wide variety of metabolic and structural changes in the cell. Although “regulate” and “affect” are difficult to be distinguished *in vitro*, it must be kept in mind that they differ by specificity, exclusivity, sensitivity, reversibility, profoundness and, particularly, feasibility for intervention.

Nevertheless, the strongest and direct blockage for the proposed “Ca^2+^-dependent α-secretase” is the current “Ca^2+^ overload” dogma, since the former concept points to a Ca^2+^ signal deficiency in the aging brain, whereas the latter implicates “excessive” signals there – two opposing and uncompromising intervention approaches on a *central* drug target.

It is thus clear that this dogma is at the epicenter of the gridlocks. By overlooking the energy-dependent and dynamic wave natures of Ca^2+^ and by overly relying on its static changes in the cell-death stage, this dogma lacks scientific rigor and panoramic vision on the *aging-to-sAD* progression, yet being inappropriately promoted by an NIA policymaker (Chen et al., 2011b). It thus has authoritatively obstructed the understanding of the central role of age-related Ca^2+^ *early and normal* changes in Aβ overproduction (and also in tangle formation and cognitive decline; see below).

So, NIA faces hard options today: to revisit the dogma, or to let the gridlocks to stay. This is a difficult but lifeline decision that NIA and the field must make.

## APP Mutations Disturb α-Processing, Not β-/γ-Processing

There are other interesting science issues to discuss. One of them is: how do pathogenic APP mutations cause Aβ increase in early onset familial AD (EOAD)? These mutations involve three groups: Swedish, London, and Dutch, which are long believed “to render β- or γ-processing more efficient” (except for Dutch mutations; Bagyinszky et al., 2014). But, this model is questionable now because of the multiplicity of “β-/γ-secretases” and the rarity of the mutations that make enzymatic reactions more efficient (see above).

Upon a closer look at the issues, I now think it more likely that these mutations increase Aβ by *decreasing* α*-processing*. For one thing, this can explain the sources of the additional full-length APPs. But is it reasonable?

**Figure 4:**
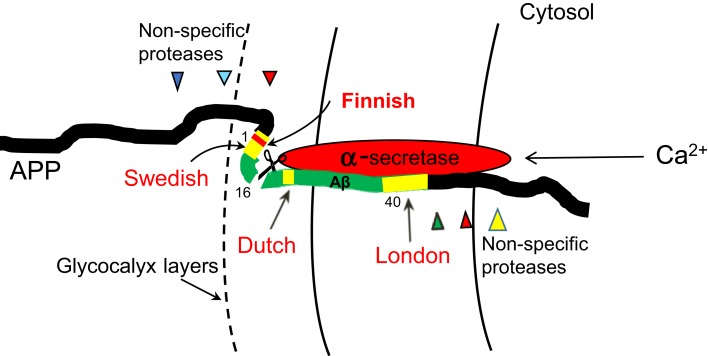
**A proposed model for how APP mutations disturb α-processing**. No doubt, Dutch mutations can directly do so. London mutations may also change the conformation of APP and disturb its binding to α-secretase in a crowed membrane environment, thus reducing α-processing. Swedish mutations, though at outside of the membrane, are embedded in the glycocalyx layers at the immediate cell surface where the movement of this section of APP relative to α-secretase is hindered, thus allowing the mutations to affect α-processing. The Swedish mutations may also be made closer to α-secretase by APP self-folding. A protective “Finnish” mutation is also depicted.

No doubt, the model can directly explain the roles of the Dutch mutations, which change the conformation of APP at a very close vicinity to the α-cleaving site, thereby robustly disrupting the interaction of APP with α-secretase (Figure 4). But, are other mutations too far to do so?

It must be noted that the relative movements of APP and α-secretase are constrained in the membrane. Such a crowed microenvironment means that the conformational changes of APP caused by London mutations may also perturb the binding of APP to α-secretase, thus reducing the α-cleavage. But due to their farther distances to the α-site, London mutations would be expected to have less potent effects than Dutch mutations. Indeed, Dutch mutations cause dramatic Aβ deposits on the blood vessels, leading to hemorrhage and early death of the carriers (Levy et al., 1990; Hendriks et al., 1992), conditions that are much severer than those caused by London mutations.

Swedish mutations, on the other hand, are located at outside of the cell, but may not move freely either, because the immediate surface of the cell is embedded in the glycocalyx layers (glycoproteins and polysaccharides). This may allow the conformational changes brought by Swedish mutations in this section of APP to also interfere with α-cleavage (Figure 4).

By contrast, if β- and γ-secretases are really “rate-limiting” in Aβ genesis and their cleavages are really made “more efficient” by the mutations, then Dutch mutations, due to their farther distances to either the β- or γ-cleaving site than London and Swedish mutations, would be expected to be the least potent in overproducing Aβ – contrary to the observations.

Also interesting is that a protective mutation has recently been found within the Swedish area (“Finnish mutation” A673T; Jonsson et al., 2012) (Figure 4). I think that it may represent a rare “gain-of-function” mutation that somehow enhances the interaction of α-secretase and APP thus decreasing Aβ.

## Why are Tau Fragments Much Longer than Aβ?

The mechanism of origins of neurofibrillary tangles is unknown today, despite intensive studies. While most current studies assume an “overactivation” of protein kinases as the initial cause (Cruz and Tsai, 2004; Medina et al., 2011), we have proposed a new model from a perspective of “inefficient calpain and calcineurin” (the reasons for why not kinases nor other proteases and phosphatases involved were discussed elsewhere; Chen and Nguyen, 2014).

**Figure 5:**
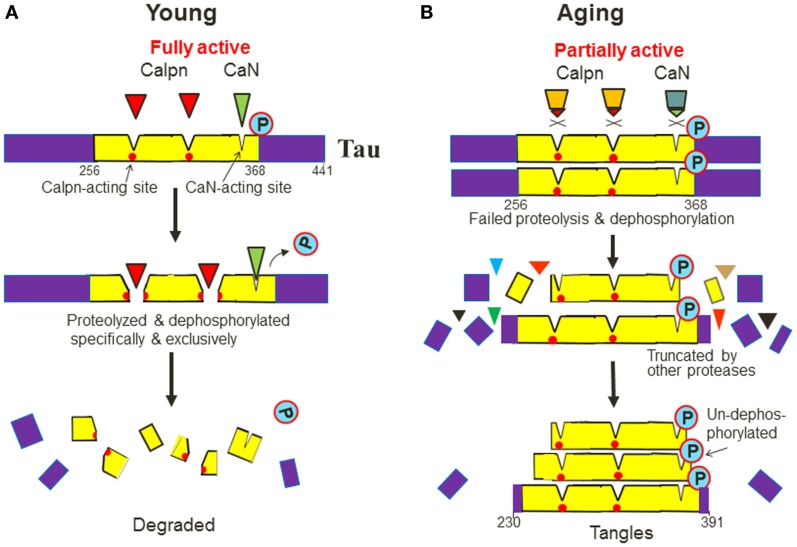
**A new model for how PHF tau is formed, why it is much longer than Aβ and is also phosphorylated**. **(A)** Tau contains multiple calpain (Calpn)-acting sites and at least one calcineurin (CaN)-acting site within its four-repeat regions (from residue 256 to 368). Ca^2+^-dependent calpain and calicineurin recognize tau in a specific and exclusive manner and completely proteolyze and dephosphorylate tau in the young. **(B)** During aging, however, the two enzymes are inefficient so tau will accumulate and will not be attacked by any other proteases and phosphatases at the same sites but will be truncated alternatively by many non-specific proteases at other sites. Thus, their core fragments containing the calpain- and calcineurin-acting sites will be spared and deposited with varying lengths that are much longer than Aβ, and also stay un-dephosphorylated, or seemingly “hyperphosphorylated”, in the aging brain.

Now, I also modified this model slightly to further explain why the deposited tau (PHF tau) is much longer than Aβ (Figure 5). PHF tau is known to be truncated at both ends and the resulting fragments start from around residue 230 to 391 (deduced from the antibody-recognizing epitopes in the longest tau isoform 441; Guillozet-Bongaarts et al., 2005; Iqbal et al., 2010). This region harbors four microtubule-binding repeats (from residues 256 to 368). But why is PHF tau so much longer (about 160 amino acids) than Aβ?

This may be because that tau, a calpain substrate, contains several calpain-cleaving sites. Indeed, it has been suggested that PHF tau contains as many as nine predicted calpain-cleaving sites (Yang and Ksiezak-Reding, 1995). Since PHF tau is phosphorylated, at least one calcineurin-acting site should reside in this region and the site should also be *exclusively* accessible by calcineurin (Figure 5A). So, after both calcineurin and calpain become inefficient during aging, tau will not be attacked by any other phosphatases or proteases at the two enzymes’ native acting sites, but are truncated into fragments that encompass those sites. Thus, these fragments would stay *un-dephosphorylated* (Figure 5B). Indeed, the term “un-dephosphorylated” is more accurate than “hyperphosphorylated,” which proactively implies an “overactivated” process. In addition, the model also predicts that the PHF tau fragments will have varying lengths at their both ends, similar to Aβs.

Of particular interest is that many pathogenic gene mutations in tau have been found to cause severer and earlier tangle formation in frontotemporal dementias (FTDs), but the mechanism involved is unknown. Now, I notice that a vast majority of these mutations are located within or near the predicted calpain/calcineurin-acting region (Wolfe, 2009). Thus, it is reasonable to believe that the mutations cause tangle formation mainly by changing the conformation of tau, thereby reducing its sensitivity to calpain and calcineurin. This reinforces the central roles of Ca^2+^ signaling and two Ca^2+^-dependent enzymes in the tangle formation.

Alternative to this model, other proteases (e.g., caspases and proteasomes) and phosphatases (e.g., PP2A, PP1A, PP1B and tyrosine phosphatase) have also been suggested to be responsible for PHF tau formation. These models need to explain: (a) how PHF tau, if produced by them, can stay and be protease-/phosphatase-double resistant; and (b) why it is *selectively* deposited among many other proteins (protein “misfolding" or aggregation may not explain it; see above).

## sAD vs. EOAD: Two Different Diseases or “the Same Disease”?

The mutation-initiated mechanisms for plaques and tangles in EOAD/FTDs (by changed APP and tau conformations, the *accidental* events) contrast sharply with the aging-initiated mechanisms for the “same” plaques and tangles in sAD (by changed protease activity, a *natural* event as age advances).

This clearly indicates that sAD differs fundamentally from EOAD by origin, and indeed, they can be clinically distinguished by age 60 and eventual prevalence (< or > 50%), two defining criteria that separate senile disorders and discrete diseases (Chen et al., 2011a). This assertion thus challenges the NIA perception that EOAD and sAD are “the same disease,” the centerpiece in the “discrete disease” definition for sAD (Khachaturian, 2008).

Notably, this NIA perception is responsible for the conundrum of APP processing today. It should be pointed out that the “two competing pathways” model (Figure 1), if used only for EOAD, is correct in essence, since APP/PS mutations do change the balance of the two pathways *at the same time* (in the young mutant hosts) and they are indeed *independent* of each other (normal vs. mutation). So, the only problem is that a correct model in one disease has been incorrectly used in another disease (Selkoe, 2005) – a serious consequence of “the same disease” perception.

Another one of the many far-reaching consequences of the NIA perception is that it promotes the wide-spread use of the mutant-based animal models today for screening and testing drugs that are mostly intended for sAD intervention. But such models mimic the conditions of EOAD, not sAD. To this end, I proposed that the best animal model that mimics the sAD conditions should be the *oldest wild-type animals* tested in comparison with young animals (Chen, 1998b).

My underlying reason for this proposal is that sAD study should be essentially the *aging study* (i.e., comparing old with young), thereby revealing age-related *normal* defects for intervention, a commonsense in the studies of senile osteoporosis and atherosclerosis (targeting bone loss and slowed-digestion of cholesterol at their early phases). Unfortunately, most studies on sAD today are instead the “disease study” (comparing patients with healthy), thus uncovering numerous “aberrant pathways” in the later or cell-death stages, but too late to have any preventive values (Chen et al., 2011b).

## Conclusion

Plaques and tangles are the products of aging, similar to cholesterol or mineral deposits, at least at their initial phases. Had this commonsense been kept in mind, sAD would not have been such a super-complex disease today.

But, sAD study has not been guided by commonsense conceptions. Its “curable disease” definition – a tempting optimism but beyond what science can prove or deliver – preconditions us to view plaques or tangles as its “culprits,” or products of “overactivated” proteases and kinases. So their inhibitors must cure the disease – a point of no return with repetitive trial failures but determinedly ongoing – an unfalsifiable faith that will not fade anytime soon (Selkoe, 2011; De Strooper, 2014).

At the same time, the “Ca^2+^ overload” dogma blocked the understanding of the role of Ca^2+^ in the formation of plaques and tangles. This, in turn, has forced the field to turn to myriad other factors: β- and γ-secretases, nicastrin, Pen2, Aph-1, metalloproteases, cholesterol, CDK, GSK, p35, p25, and the list still expanding.

Such scattering studies would render the origin of plaques and tangles a super-complex and ever diverging – thus hopeless – maze, since a healthy research trend must be *converging* over time (narrowing down the suspects). This may be why there has been a deepening dissociation between paper publication and understanding of sAD. As a matter of fact, Ca^2+^ is a *single* central regulator in cognition – or “Achilles’ heel” of sAD – so studying any other factors may only address secondary or non-central issues in the disease (akin to study diabetes but not focusing on insulin-centered pathways, or study atherosclerosis not focusing on lipid metabolisms).

I, therefore, call for a game change in sAD study, particularly in the areas that are contaminated by vested interests. This issue awaits the decision of NIA and all researchers. Ignoring or delaying this long overdue decision will further hamper the rational intervention in sAD, a mission that has been delayed for 40 years.

## Conflict of Interest Statement

The author declares that the research was conducted in the absence of any commercial or financial relationships that could be construed as a potential conflict of interest.
